# 1951. Guiding COVID-19 Booster Vaccinations by COVID-19 Quantitative Spike Ig Antibody Titers Regardless of HIV Status, Immunosuppression, and Age

**DOI:** 10.1093/ofid/ofac492.1578

**Published:** 2022-12-15

**Authors:** Ricky K Hsu, Laurence Brunet, Jennifer S Fusco

**Affiliations:** AIDS Healthcare Foundation\ NYU School of Medicine, New York, New York; Epividian, Inc., Durham, North Carolina; Epividian, Inc., Durham, North Carolina

## Abstract

**Background:**

In-vitro neutralizing antibody (Ab) titers correlated with ∼250 IU/mL Spike Ig Ab level for the Delta COVID-19 variant, establishing the 2021 French and Swiss cutoff for booster guidance. In a New York City healthcare clinic where those guidelines were adopted, we aimed to quantify vaccination responses in HIV+ and HIV- individuals to assess the utility of quantifying antibodies to guide booster timing.

**Methods:**

Adults who were fully vaccinated against SARS-CoV-2 virus (i.e., 2 Pfizer, 2 Moderna or 1 J&J vaccine) were included if >1 Roche SARS-CoV-2 Semi-Quant Spike Ig Ab test was performed >21 days after vaccination and before any booster (through 03DEC2021). Vaccine response was assessed at the first Ab test and considered adequate (>250 IU/mL) or inadequate (low: ≥51 to ≤250 IU/mL; no response: < 51 IU/mL). The rate of Ab decline was estimated with linear regression, using all sequential Ab tests over the first 6 months between vaccination and boosting. Analyses were stratified by vaccine type, HIV status and CD4 count in HIV+ ( >200 cells/µL cutoff).

**Results:**

Out of 1979 patients, 869 completed their primary vaccinations, of whom 825 (95%) had ≥1 eligible Ab test (HIV+: 512; HIV-: 313; Table). Overall, 83% had an adequate immune response to vaccination (Pfizer: 82%, Moderna: 94%, J&J: 51%), with similar findings regardless of HIV status and CD4 count (Figure 1). In those with ≥2 Ab tests within six months between vaccination and boosting, Ab levels declined at a rate of 91 IU/mL per month (95% CI: -138, -44). While some variation was observed, rates of Ab decay were generally consistent across vaccine, HIV status and CD4 count strata (Figure 2). Only 1/7 breakthrough COVID-19 infections occurred post booster (6 days later).

**Figure:**
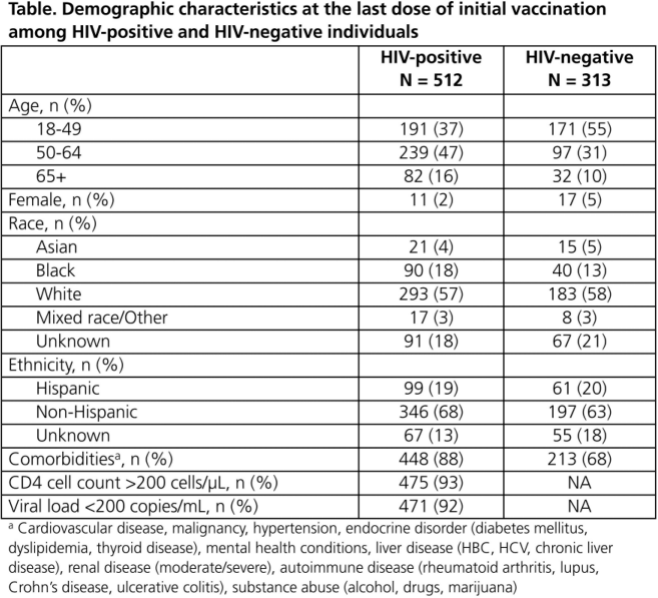

**Figure:**
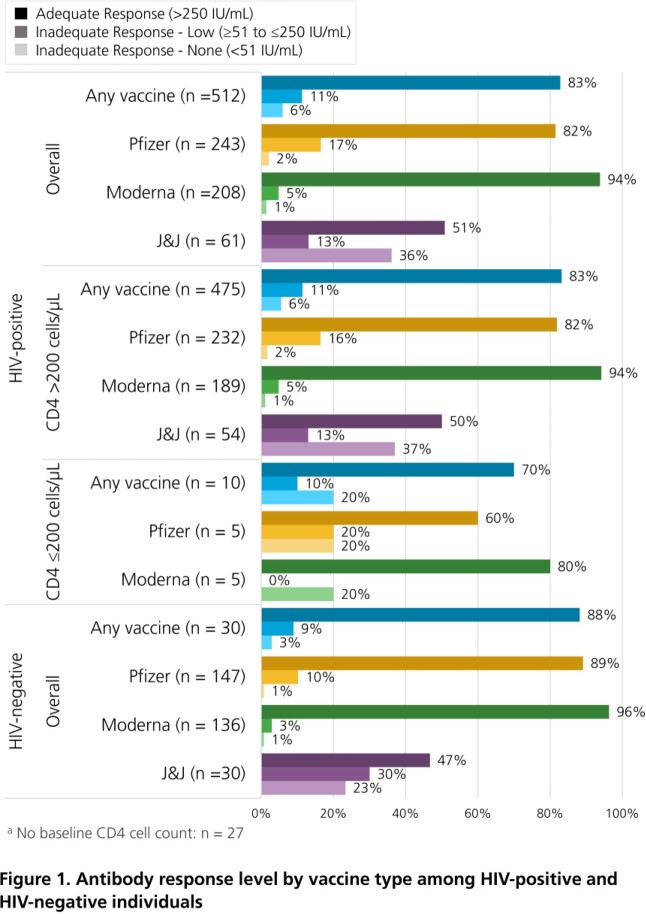

**Figure:**
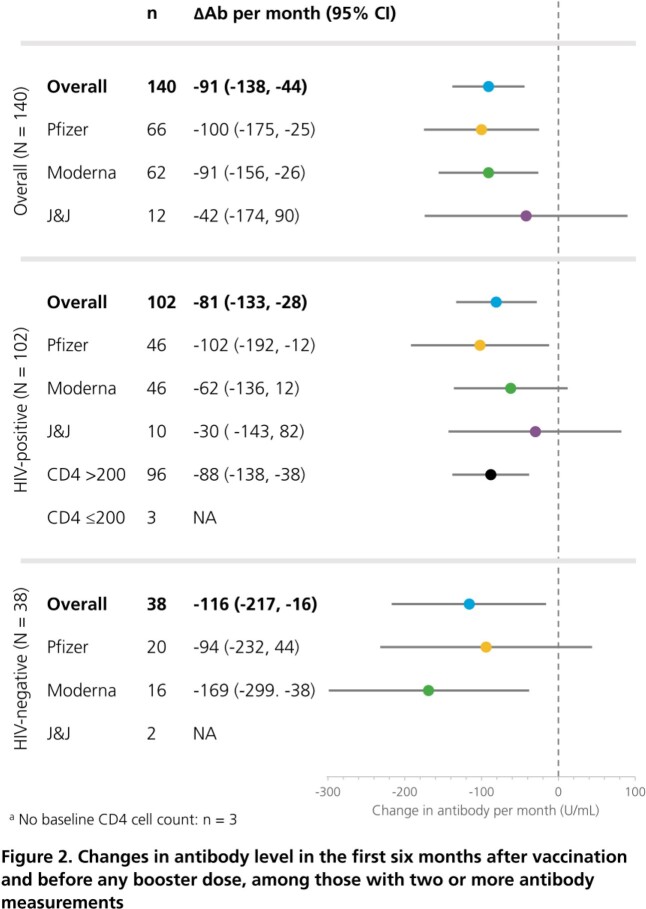

**Conclusion:**

In the pre-omicron era, primary COVID immunization with a mRNA vaccine generally yielded adequate Ab responses, although inadequate responses were observed in 19% of Pfizer, 6% of Moderna, and 49% of J&J vaccine recipients. Ab levels decreased at an average rate of 91 IU/mL per month after primary immunization. Variability in vaccine responses and Ab declines show the utility of measuring spike Ig Ab levels rather than using empiric time frames for booster guidance. Omicron-specific quantitative IgG neutralization levels must be established to inform preventative care.

**Disclosures:**

**Ricky K. Hsu, MD**, Gilead: Honoraria|Merck: Honoraria|ViiV: Advisor/Consultant|ViiV: Grant/Research Support|ViiV: Honoraria **Laurence Brunet, PhD**, AIDS Healthcare Foundation: Client of my employer|EMD Serono: Client of my employer|Gilead Sciences: Client of my employer|Janssen: Client of my employer|Merck & Co: Client of my employer|TheraTechnologies: Client of my employer|ViiV Healthcare: Client of my employer **Jennifer S. Fusco, BS**, AIDS Healthcare Foundation: Client of my employer|EMD Serono: Client of my employer|Gilead Sciences: Client of my employer|Janssen: Client of my employer|Merck & Co.: Client of my employer|TheraTechnologies: Client of my employer|ViiV Healthcare: Client of my employer.

